# Exposure to Internet-Based Tobacco Advertising and Branding: Results From Population Surveys of Australian Youth 2010-2013

**DOI:** 10.2196/jmir.5595

**Published:** 2016-06-23

**Authors:** Sally Dunlop, Becky Freeman, Donna Perez

**Affiliations:** ^1^ Division of Cancer Screening and Prevention Cancer Institute New South Wales Sydney Australia; ^2^ Sydney School of Public Health University of Sydney Sydney Australia; ^3^ Prevention Research Collaboration Sydney School of Public Health University of Sydney Sydney Australia; ^4^ Charles Perkins Centre University of Sydney Sydney Australia

**Keywords:** youth, tobacco, social media, advertising

## Abstract

**Background:**

Since legislation prohibiting tobacco advertising in traditional media, online communication platforms and social media have become one of the few avenues for the tobacco industry to promote its products to Australians. Little is currently known about the exposure of young people to these new media promotions.

**Objective:**

To measure exposure to Internet-based tobacco advertising and branding among Australian youth, identify common formats of branding encountered, and examine the association between exposure and smoking susceptibility.

**Methods:**

The Tobacco Promotion Impact Study is a repeat cross-sectional telephone survey of young people (12-24 years) in 2 Australian states, conducted yearly from 2010 to 2013 (total n=8820). The survey included questions about past-month exposure to Internet-based tobacco advertising and tobacco company branding. Changes in levels of exposure, characteristics of exposed youth, and the association between exposure and smoking susceptibility were explored.

**Results:**

Past-month exposure to Internet-based tobacco advertising and branding among young people increased over the years of the survey (advertising: 21% in 2010 to 29% in 2013; branding: 20% in 2010 to 26% in 2013). The participants who were younger, female, from lower socioeconomic status, and never-smokers were more likely to report exposure. Facebook was the most commonly cited platform for encountering tobacco branding in 2013 (22% of all branding). Compared with young people interviewed in 2013, participants in 2010 were significantly less likely to report exposure to tobacco branding on social media (odds ratio [OR] 0.26, 95% CI 0.20-0.33, *P*<.001) or 2011 (OR 0.46, 95% CI 0.37-0.57, *P*<.001). Among never-smokers aged 12-17 years, exposure to online advertising and branding (OR 1.32, 95% CI 1.11-1.57, *P*=.002) or branding alone (OR 1.39, 95% CI 1.10-1.77, *P*=.007) were significant predictors of smoking susceptibility.

**Conclusions:**

Ensuring tobacco advertising bans are inclusive of Internet-based media is essential. Given the global nature of Internet-based content, cooperation among signatory nations to the World Health Organization Framework Convention Alliance on Tobacco Control will be necessary.

## Introduction

It is well established that the implementation of a comprehensive tobacco advertising ban is a crucial element of effective tobacco control [1]. Legislation that only limits certain types of tobacco advertising, promotion, and sponsorship enables the tobacco industry to shift resources to unregulated forms of marketing [2]. Equally, the growth in the number and accessibility of new media channels creates opportunities for the tobacco industry to promote its branding and products. The accelerated uptake of communications technology, particularly the use of online social media platforms such as Facebook, has fueled calls for increased surveillance of online tobacco advertising and improved knowledge of the impact it may be having on consumers [3].

The emerging body of research examining prosmoking imagery and advertising online is primarily descriptive in nature [4]. Case study [5,6] and content analysis research [7-9] of new media have shown a proliferation of prosmoking messages, imagery, and tobacco brand promotion. Although direct promotion of tobacco via advertisements is not permitted by the owners of many social media sites (eg, Facebook [10]), the potential for tobacco companies to use these sites to raise the visibility of their products and promote tobacco use remains [11-13]. Tobacco companies can still operate branded pages and channels on social media portals, either directly or through advertising firms that can include product updates, images, videos, and links to real-life events. A recent analysis of 70 cigarette brands on social media revealed more than 120,000 video clips on YouTube and 238 Facebook fan pages with more than 1 million “likes,” indicating high user interaction [14].

Given the explosive growth in social media use coupled with the ubiquitous uptake of Internet-enabled mobile phones [15,16], determining levels of exposure to this type of tobacco promotion is essential if tobacco advertising bans are to keep pace with modern marketing methods. Although previous research has revealed that both young people [17] and adults [18] are regularly exposed to Internet-based tobacco advertising, very little published research has determined the level of exposure occurring on popular social media websites. Data collected from US school students in 2011 showed that 11% of youth had received advertisements or promotions from tobacco companies via Facebook or Myspace [19]; however, this does not capture the more indirect forms of promotion that are common on social media.

The potential impact of exposure to online tobacco advertising and promotion on youth is of particular concern. US data show that 92% of teenagers aged 13-17 years are online daily, with 24% reporting they are online almost “constantly.” Survey data from 2015 showed that Facebook is the most used social media site among American teenagers, ages 13 to 17 years, with 71% using the site [20]. Globally, Facebook is the world’s most popular social media site, with 1.55 billion monthly active users as of September 2015 [21]. Although a large body of evidence has demonstrated the link between exposure to traditional tobacco branding and smoking susceptibility [22], much remains to be learned about the effects of exposure to tobacco branding in the digital space. One study has shown that exposure to tobacco advertising via Facebook or Myspace was associated with protobacco beliefs and willingness to try smoking among young never-smokers [19], but no study to date has examined the effect of exposure to online tobacco company branding in general, which is the more common form of tobacco promotion on social media.

Australia is known as a “dark market” for tobacco products, with increasingly prohibitive advertising restrictions since the 1970s. Bans on television and radio advertisements were followed by bans on outdoor advertising and sponsorship of sporting events in the 1980s, advertising in the print media and retail point-of-sale in the 1990s, and point-of-sale tobacco displays from 2010. In 2013, Australia introduced the world-first plain packaging legislation in which all forms of branding were removed from tobacco packs in an effort to reduce one of the last forms of tobacco promotion in Australia. Although the global nature of the Internet makes regulating online advertising challenging, amendments to the Tobacco Advertising Prohibition Act made it an offense for any person to publish tobacco advertising on the Internet or other electronic media from Australia, from September 2012 [23]. These regulations also set out requirements for Internet point-of-sale advertisements: they must be in a plain, text-only format with no product images, inclusive of graphic health warnings and accompanied by warnings about age restrictions on sales. No research to date has investigated Australians’ exposure to online tobacco advertising and branding in the context of these evolving restrictions.

The primary objective of this study was to assess the exposure of Australian youth to online tobacco advertising and promotion and determine whether exposure has changed in recent years in relation to changes in opportunities for tobacco promotion (outlined in Table 1). We sought to measure exposure to online tobacco advertisements, as well as to more general tobacco company branding, and we profiled youth most likely to be exposed. Additionally, we tracked any changes in the locations where branding was encountered, including social media sites. Finally, we aimed to determine if the established association between exposure to tobacco marketing and smoking susceptibility among youth [24] was also evident with exposure to online tobacco advertising and tobacco branding.

**Table 1:** Timing of restrictions on tobacco advertising and promotions in relation to Tobacco Promotion Impact Study.

Year	Month	TPIS^a^ waves	Restrictions
2010	January		Point-of-sale display ban – NSW^b^ large retailers
	June	Wave 1	
	July		Point-of-sale display ban – NSW small retailers
2011	June	Wave 2	
	November		Point-of-sale display ban – QLD^c^ all retailers
2012	June	Wave 3	
	September		Internet advertising ban
	October		Plain packaging introduced
2013	June	Wave 4	

^a^TPIS: Tobacco Promotion Impact Study.

^b^NSW: New South Wales.

^c^QLD: Queensland.

## Methods

Data for this study come from the Tobacco Promotion Impact Study (TPIS), conducted in the Australian states of New South Wales (NSW) and Queensland (QLD). The study has a repeat cross-sectional design with yearly telephone surveys conducted in June of each year from 2010 to 2013 (total n=8820). The TPIS monitors adolescents’ and young adults’ (12-24 years) exposure to tobacco promotions in a range of places, as well as smoking-related cognitions and behaviors. Households were recruited using random digit dialing and participants within households were recruited using random selection (selecting the *n*th oldest eligible person aged 12 to 24 years). From 2010 to 2012, recruitment was conducted using landline phone numbers only. In 2013, because of concerns about the increasing proportion of Australian homes without a landline phone number (from 17% in 2010 to 22% in 2012) [25], a supplemental sample of participants was also recruited through random-digit dialing to mobile phone numbers. Use of this supplemental sample is described below. Permission was obtained from parents of 12- to 15-year-olds before conducting each interview. Cooperation rates averaged 70% among eligible respondents. When taking into account households of unknown eligibility, response rates averaged 42% (American Association for Public Opinion Research Response Rate #3) [26]. The study was approved by the NSW Population and Health Services Research Ethics Committee.

### Measures

#### Exposure to Internet-Based Tobacco Promotion

To take into account direct advertising as well as the forms of more indirect promotion encountered on social media sites, exposure to both online tobacco advertising and online tobacco branding was assessed. All respondents were asked, “In the past month, how often have you seen any promotions or advertising for cigarettes or other tobacco products in the following places?”. The list of possible places included “the Internet” (online advertising). They were also asked, “In the past month, how often have you seen cigarette brands, tobacco company names, or logos on the internet?” (online branding). Responses to both questions were “never,” “rarely,” “sometimes,” and “often.” Responses to both questions were dichotomized because of negative skew (0=never or rarely vs 1=sometimes or often). Responses were also combined to indicate whether an individual was (1) never/rarely exposed to advertising or branding, (2) sometimes/often exposed to both advertising and branding, (3) sometimes/often exposed to advertising only, or (4) sometimes/often exposed to branding only.

**Figure 1:**
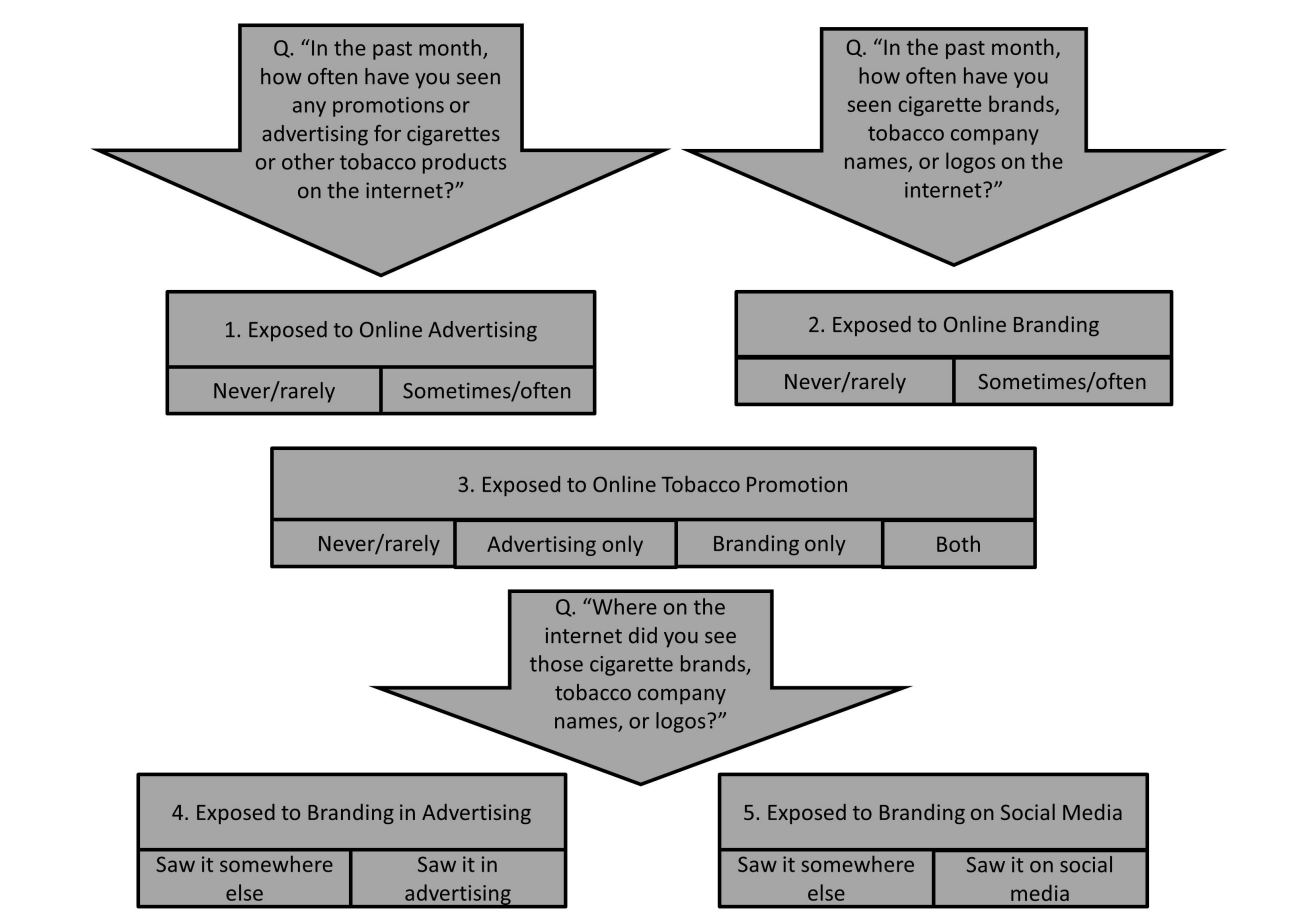
Summary of questions and derived variables relating to online tobacco promotion exposure. Survey questions are denoted by “Q”; derived variables are numbered.

In order to explore types of branding encountered, young people exposed to branding were asked in what formats the cigarette brands, tobacco company names, or logos were encountered on the Internet. Responses were recorded verbatim and matched to a list of possible websites or types of online advertising. We also combined responses in order to report on the proportion of respondents seeing (1) branding in advertisements (pop-up advertisements, banner advertisements, Google advertisements, website advertisements); (2) branding on social media (Twitter, Facebook, YouTube, Myspace); (3) branding in personal communications (email, instant messenger, forums); and (4) branding on content-controlled websites (news sites, sports sites, blogs, gaming sites, Yahoo, Ninemsn; see Figure 1).

#### Current Smoking

Respondents were asked if they had ever had a puff of a cigarette, how many cigarettes they had smoked in their lifetime, and if they had smoked in the past month. Based on stage models of smoking uptake [27], they were classified as follows: (1) never-smokers (never taken a puff); (2) experimenters (smoked less than 5 cigarettes ever, or smoked 5-100 cigarettes in their lifetime but not in the past month); (3) current smokers (smoked more than 5 cigarettes in their lifetime, and smoked in the past month); or (4) ex-smokers (smoked more than 100 cigarettes in their lifetime but not in the past month).

#### Smoking Susceptibility

Never-smokers were asked a series of validated questions to determine their susceptibility to smoking in the future [28,29]. Participants were classified as nonsusceptible if they answered “definitely no” to each of the following questions: “Do you think that you will try cigarettes sometime soon?”; “Do you think you will smoke a cigarette sometime in the next year?”; and “If a friend offered you a cigarette, would you try it?” (response options: 1=definitely no, 2=probably no, 3=probably yes, 4=definitely yes). Participants who did not answer each of those questions with “definitely no” were classified as susceptible.

#### Smoking Exposure

Respondents reported on the number of current smokers in their household and how many of their five closest friends smoked.

#### Average Daily Internet Use

Respondents were asked, “How much time do you spend on average per day on the Internet, if at all?” Responses were recorded in minutes and divided by 60 to represent hours per day.

#### Demographics

Age, sex, state of residence, and year of interview were included. Postcodes were used with the Socio-Economic Indexes for Areas (SEIFA) [30] to indicate low (quintiles 4-5) or moderate-high (quintiles 1-3) socioeconomic status (SES).

### Statistical Analysis

We first conducted logistic regression analyses to explore changes over time in exposure to (1) online tobacco advertising and (2) online tobacco branding. Each logistic regression model included year of interview, demographics (age, sex, SES, state), Internet use, smoking exposures (friends, household), and smoking status as predictors. Because of the low number of ex-smokers in the sample, and the similar demographic profile of ex-smokers and current smokers, these groups were combined for these analyses. These models also identified individual characteristics of exposed youth.

Next, we examined changes over time in the format that tobacco branding was encountered online. The overall number of young people who reported seeing each of the branding formats was relatively small (ranging from 1 to 829), so we report only on those mentioned by at least 5% of the sample who recalled seeing branding. We used Pearson chi-square tests to detect significant differences in exposure to specific branding formats, as well as the types of branding, over survey years. The overall numbers of young people who reported seeing branding in personal communications (n=90) or on content-controlled websites (n=226) were small, therefore they were not investigated further. Logistic regression analyses were conducted to predict encountering branding through (1) advertisements and (2) social media. Year of interview, demographics, smoking status, smoking exposures, and Internet use were entered as predictors.

Finally, we explored whether smoking susceptibility was associated with exposure to online advertising and tobacco branding. As well as testing whether smoking susceptibility was associated with exposure to online advertising, we were also interested in whether exposure to tobacco company branding in the absence of advertising would be associated with susceptibility. Therefore, we created a 3-level variable classifying participants as having been exposed in the past month (1) never/rarely to online tobacco advertising or branding, (2) sometimes/often to tobacco advertising (with or without branding), or (3) sometimes/often exposed to tobacco company branding but not advertising. This variable was entered as a predictor in a logistic regression model predicting smoking susceptibility, with demographic characteristics, year of interview, smoking exposures, and Internet use as covariates. Because the established link between exposure to protobacco marketing and smoking susceptibility applies primarily to adolescents [24], we conducted this analysis separately for 12- to 17-year-olds and 18- to 24-year-olds.

The supplemental mobile phone sample was added in 2013 to assess whether any changes in outcomes between years of the survey were due to changes in the characteristics of the population covered by landlines. Previous studies have found that adding a mobile component to a landline population survey gives a more representative sample [31], but it also has the potential to result in changes to population estimates that are a consequence of the design change, rather than a real change [32]. Comparing both the landline-only and the dual-frame (landline and mobile) samples with previous years’ samples allows this issue to be explored. Therefore, all analyses in this study were conducted twice. The first set of analyses used the landline sample only, comparing differences between years while minimizing bias due to changes in sampling. The second set of analyses used the dual-frame sample for 2013; comparing differences between years while minimizing the influence of the changing composition of a sample recruited via landline only. The results from the second set of analyses are only reported when the pattern of results differ from the first.

The gender distribution of this sample was relatively consistent with population parameters as defined by Australian Bureau of Statistics data [30]. There were, however, some discrepancies in the age distribution, particularly a slight overrepresentation of 16- to 19-year-olds but underrepresentation of 20- to 24-year-olds. Given these discrepancies, data were weighted to the NSW and QLD populations of 12- to 24-year-olds for age, sex, and region distributions from Census data [30] using poststratification weights. In the set of analyses including the 2013 mobile phone supplement, additional weighting was used to account for telephone status (landline only, mobile phone only, or dual user). All analyses were conducted using Stata v11.1 [33].

## Results

Sample characteristics and exposure to online tobacco promotion for each survey wave are listed in Table 2(with landline and dual-frame samples for 2013 shown separately). The samples in each year of the survey were similar in terms of age and sex. There was a significant difference in SES, with the highest proportion of respondents from a moderate-high SES area in 2011. There was a significant difference in smoking status in the landline sample: current smoking decreased from 16% to 12% over the years of the survey. In the dual-frame sample, there was an increase from 12% in 2012 to 16% in 2013. Similarly, the number of smoking friends and household members also decreased significantly in the landline sample but increased in the dual-frame sample in 2013. Average daily Internet use increased significantly, from 2.43 hours in 2010 to 3.28 hours in 2013 (3.33 in the dual-frame sample).

**Table 2:** Sample characteristics.

Characteristic		2010 (n=2000)	2011 (n=2010)	2012 (n=2003)	2013 landline (n=2001)	*P*^a^	2013 dual-frame (n=2807)	*P*^a^
**Age in years, N(%)**^b^								
	12-15	669 (32)	649 (32)	619 (32)	684 (31)		833 (31)	
	16-19	826 (31)	833 (30)	855 (30)	799 (31)		1046 (31)	
	20+	505 (37)	528 (38)	529 (38)	518 (39)	.974	928 (39)	.950
**Sex, N(%)**^b^								
	Female	975 (49)	990 (49)	992 (49)	980 (49)		1325 (49)	
	Male	1025 (51)	1021 (51)	1011 (51)	1021 (51)	>.99	1482 (51)	>.99
**State, N(%)**^b^								
	NSW^c^	1000 (50)	1004 (50)	1000 (50)	1001 (50)		1407 (50)	
	QLD^h^	1000 (50)	1000 (50)	1000 (50)	1000 (50)	>.99	1400 (50)	>.99
**SES**^d,f^ **, N(%)**^b^								
	Low	557 (28)	497 (25)	578 (29)	536 (26)		735 (28)	
	Moderate-high	1443 (72)	1514 (75)	1425 (71)	1465 (74)	.011	2056 (72)	.015
**Smoker, N(%)**^b^								
	Current	293 (16)	243 (13)	220 (12)	207 (12)		369 (16)	
	Never	1178 (56)	1278 (61)	1276 (61)	1376 (64)		1769 (60)	
	Former	53 (4)	42 (3)	36 (2)	36 (3)		70 (3)	
	Experimenter	476 (25)	448 (24)	471 (25)	382 (21)	<.001	599 (22)	<.001
**Internet-based tobacco promotion exposure, N(%)**^b^								
	Never/rarely	1330 (70)	1273 (66)	1200 (62)	1158 (61)		1651 (59)	
	Ads^g^ and branding^e^	218 (11)	266 (13)	313 (15)	335 (16)		442 (16)	
	Ads only^e^	208 (10)	240 (12)	245 (12)	272 (13)		395 (15)	
	Branding only^e^	200 (10)	205 (10)	223 (11)	215 (10)	<.001	293 (11)	<.001
Friends who smoke, mean (SD)		1.27 (1.60)	1.10 (1.51)	1.05 (1.47)	0.93 (1.41)	<.001	1.11 (1.52)	<.001
Household members who smoke, mean (SD)		0.52 (0.86)	0.47 (0.86)	0.49 (1.01)	0.42 (0.82)	.048	0.51 (0.99)	<.001
Internet use in hours, mean (SD)		2.43 (2.36)	2.63 (2.49)	2.90 (2.59)	3.28 (2.96)	<.001	3.33 (3.00)	<.001

^a^*P* values from chi-square tests for differences between proportions or analysis of variance tests for differences between means.

^b^Numbers are unweighted, percentages are weighted.

^c^NSW: New South Wales;.

^d^Based on postal code.

^e^Sometimes/rarely exposed.

^f^SES: socioeconomic status

^g^ads: advertisements.

^h^QLD: Queensland.

### Exposure to Internet-Based Tobacco Advertising and Branding

There were significant differences in recent exposure to Internet-based tobacco promotion across the years of the survey, with the proportion of the sample never or rarely exposed decreasing from 70% in 2010 to 61% in 2013; 59% in dual-frame sample. In 2013, 16% of participants were recently exposed to both advertising and branding, 13% exposed to advertising only; 15% in dual-frame sample, and 10% to branding only; 11% in dual-frame sample. 

Table 3 shows the proportions of youth exposed to Internet-based tobacco advertising and branding, along with the results from the logistic regression analyses predicting exposure in the landline samples. Controlling for demographic and smoking characteristics, youth interviewed in 2010 or 2011 were significantly less likely to have recently been exposed to Internet-based tobacco advertising than those interviewed in 2013. In the landline sample, there was no significant difference in the likelihood of being exposed to advertising or branding between 2012 and 2013. However, in the dual-frame sample, youth interviewed in 2012 were significantly less likely than those interviewed in 2013 to report exposure (odds ratio [OR] 0.85, 95% CI 0.73-0.98, *P*=.024). In the model predicting exposure to tobacco company branding, youth interviewed in 2010 and 2011 were significantly less likely to report recent exposure than those interviewed in 2013. There was no change in the proportion exposed to branding between 2013 and 2012 in either the landline or dual-frame samples.

**Table 3:** Proportions of youth (unadjusted) with exposure to Internet-based tobacco advertising and branding, and results from logistic regression analyses predicting exposure (results from landline sample).

Characteristic		Exposed^a^ to tobacco advertising/promotion (n=7856)					Exposed^a^ to tobacco company branding (n=7858)				
		%^b^	OR^c,d^	95% CI		*P*	%^b^	OR^c^	95% CI		*P*
**Year**											
	2010	21	0.66	0.57	0.77	<.001	20	0.71	0.61	0.83	<.001
	2011	24	0.81	0.70	0.94	.006	22	0.82	0.70	0.95	.010
	2012	27	0.92	0.80	1.07	.284	26	0.98	0.84	1.14	.780
	2013	29	(ref^e^)				26	(ref)			
**Age, years**											
	12-15	33	2.12	1.81	2.49	<.001	32	2.58	2.18	3.04	<.001
	16-19	27	1.61	1.40	1.86	<.001	25	1.79	1.54	2.08	<.001
	20+	17	(ref)				15	(ref)			
**Sex**											
	Female	29	(ref)				26	(ref)			
	Male	22	0.71	0.64	0.79	<.001	21	0.77	0.69	0.86	<.001
**State**											
	NSW^f^	26	(ref)				23	(ref)			
	QLD^g^	25	0.96	0.87	1.07	.499	24	1.05	0.94	1.18	.348
**SES**^h,j^	Low	28	(ref)				27				
	Moderate-high	24	0.83	0.74	0.94	.003	22	0.81	0.72	0.92	<.001
**Smoking**											
	Never-smoker	30	(ref)				27	(ref)			
	Experimenter	22	0.81	0.70	0.94	.006	21	0.87	0.74	1.01	.065
	Current or ex-smoker	12	0.37	0.29	0.47	<.001	13	0.44	0.35	0.57	<.001
Friends who smoke		N/A^i^	1.08	1.03	1.13	.001	N/A	1.10	1.05	1.15	<.001
Household members who smoke		N/A	1.08	1.01	1.15	.030	N/A	1.12	1.05	1.20	.001
Internet, hours		N/A	1.04	1.02	1.06	<.001	N/A	1.04	1.02	1.06	<.001

^a^Exposure=sometimes or often exposed versus never or rarely.

^b^Percentages are weighted.

^c^Odd ratios are from multivariable analyses.

^d^OR: odds ratio.

^e^ref: reference category.

^f^NSW: New South Wales.

^g^QLD: Queensland.

^h^SES: socioeconomic status.

^i^N/A: not applicable.

^j^Based on postal code.

There were many similarities in the characteristics of youth most likely to be exposed to Internet-based advertising and branding: participants who were younger, female, and from lower SES areas were more likely to report exposure. Current smokers were less likely to be exposed than never-smokers. There were positive associations between friends’ smoking, household members’ smoking, average daily Internet use, and both types of exposure.

### Format of Tobacco Branding

Across all years of the survey, when asked where they had seen tobacco company branding on the Internet, the most common answer among youth was that they did not know (Table 4). However, this proportion decreased significantly from 40% in 2010 to 28% in 2013 (29% dual-frame). In 2013, the most common place to report seeing tobacco branding was on Facebook, followed by pop-up messages, banner advertisements, YouTube, and Google advertisements. Chi-square analyses showed that exposure to branding on Facebook and YouTube increased significantly over the years of the study, while exposure to branding on Google advertisements decreased.

**Table 4:** Format of branding encountered among youth who reported seeing Internet-based tobacco branding.

Format^a^	2010 (n=850)	2011 (n=967)	2012 (n=999)	2013 landline (n=1033)	*P*^b^	2013 dual-frame (n=1384)	*P*^b^
Pop-up messages	19%	21%	20%	19%	.632	20%	.643
Banner ads^c^	16%	17%	19%	17%	.314	16%	.203
Google ads	4%	4%	7%	3%	.004	3%	.002
Facebook	9%	15%	21%	22%	<.001	22%	<.001
YouTube	2%	3%	9%	12%	<.001	11%	<.001
Don’t know	40%	32%	27%	28%	<.001	29%	<.001

^a^Only formats with at least 5% of sample naming them are included.

^b^*P* value from Pearson chi-square tests for proportions.

^c^Ads: advertisements.

The proportions of youth who saw Internet-based branding and reported that they saw it in advertising or in social media, along with the results from the logistic regression analyses predicting these exposures, are listed in Table 5 (landline sample). Controlling for differences in demographic and smoking characteristics, youth interviewed in 2012 were significantly more likely than youth interviewed in 2013 to report encountering branding in advertising. When the model was run with the dual-frame sample, youth interviewed in 2011 (OR 1.21, 95% CI 1.01-1.46, *P*=.043) and 2012 (OR 1.26, 95% CI 1.05-1.51, *P*=.014) were significantly more likely than those interviewed in 2013 to report encountering branding in advertising. Conversely, youth interviewed in 2010 or 2011 were significantly less likely to have encountered branding on social media than those interviewed in 2013 (same pattern of results obtained in the landline and dual-frame samples). Males were less likely than females to have encountered branding in advertising or social media. Current and ex-smokers were less likely than never-smokers to have encountered branding in advertising. Participants with more friends who smoke, and those with higher Internet use, were more likely to have encountered tobacco company branding on social media.

**Table 5:** Proportions of youth (unadjusted) exposed to different formats of Internet-based tobacco branding, and results from logistic regression analyses predicting exposure (results from landline sample).

Characteristic		Exposed to branding in advertising versus exposed elsewhere (n=3849)					Exposed to branding on social media versus exposed elsewhere (n=3849)					
		%^a^	OR^b,c^	95% CI		*P*	%^a^	OR^b^	95% CI		*P*
**Year**												
	2010	37	0.99	0.82	1.21	.959	10	0.26	0.20	0.33	<.001	
	2011	40	1.16	0.96	1.40	.119	17	0.46	0.37	0.57	<.001	
	2012	42	1.21	1.01	1.45	.043	27	0.83	0.68	1.01	.066	
	2013	37	(ref^e^)				31	(ref)				
**Age, years**												
	12-15	43	1.18	0.97	1.44	.105	20	1.36	1.05	1.75	.018	
	16-19	38	1.03	0.86	1.24	.724	25	1.41	1.13	1.76	.002
	20+	36	(ref)				20	(ref)				
**Sex**												
	Female	41	(ref)				23	(ref)				
	Male	37	0.84	0.73	0.96	.010	20	0.83	0.70	0.97	.022	
**State**												
	NSW^f^	39	(ref)				21	(ref)				
	QLD^g^	39	0.99	0.87	1.14	.912	22	1.02	0.86	1.20	.842
**SES**^d,h^												
	Low	39	(ref)				22	(ref)				
	Moderate-high	39	0.99	0.85	1.15	.885	22	1.03	0.86	1.24	.731	
**Smoking**												
	Never-smoker	41	(ref)				21	(ref)				
	Experimenter	38	0.99	0.83	1.19	.932	21	0.94	0.75	1.18	.613
	Current or ex-smoker	28	0.70	0.51	0.95	.021	30	1.39	0.99	1.96	.060
Friends who smoke		N/A^i^	0.96	0.91	1.02	.178	N/A	1.15	1.07	1.23	<.001
Household members who smoke		N/A	1.02	0.94	1.10	.610	N/A	1.03	0.94	1.12	.578
Internet, hours		N/A	0.99	0.97	1.02	.663	N/A	1.06	1.03	1.09	<.001

^a^Percentages are weighted.

^b^Odds ratios are from multivariable analyses.

^c^OR: odds ratio.

^d^Based on postal code.

^e^ref: reference category.

^f^NSW: New South Wales.

^g^QLD: Queensland.

^h^SES: socioeconomic status.

^i^N/A: not applicable.

### Association Between Exposure to Tobacco Advertising or Branding and Smoking Susceptibility

Results from the logistic regression analysis predicting smoking susceptibility among never-smokers are shown separately for adolescents and young adults in Table 6. For adolescents, compared with those never or rarely exposed to online tobacco promotion, those exposed to online tobacco advertising (with or without branding) as well as those exposed to tobacco company branding only were more likely to be susceptible to smoking. These effects were apparent when controlling for the influence of age, household members and friends smoking, year of interview, and average daily Internet use. For young adults, there were no associations between exposure to online tobacco promotions and smoking susceptibility. These results were the same in both the landline and dual-frame samples.

**Table 6:** Proportions of nonsmoking youth (unadjusted) susceptible to smoking, and results from logistic regression analyses predicting smoking susceptibility (results from landline sample).

Characteristic		12- to 17-year-olds (n=3377)						18- to 24-year-olds (n=1594)				
		%^a^	OR^b,c^	95% CI		*P*	%^a^	OR^b^	95% CI		*P*
**Exposure**												
	Never/rarely	23	(ref^f^)				13		(ref)			
	Exposed^d^ to online ads^g^ and branding	29	1.32	1.11	1.57	.002	16	1.30	0.92	1.83	.137
	Exposed^d^ to online branding only	30	1.39	1.10	1.77	.007	11	0.76	0.44	1.31	.315
**Year**												
	2010	24	1.00	0.79	1.25	.967	10	0.64	0.41	1.00	.049
	2011	25	1.06	0.85	1.32	.610	13	0.80	0.53	1.22	.301
	2012	29	1.22	0.98	1.52	.069	15	0.99	0.67	1.46	.955
	2013	25	(ref)				15		(ref)			
**Sex**												
	Female	23	(ref)				12	(ref)			
	Male	28	1.30	1.11	1.53	.001	15	1.43	1.05	1.93	.021
**State**												
	NSW^c^	25	(ref)				13		(ref)			
	QLD^h^	26	0.99	0.85	1.16	.931	14	1.11	0.82	1.51	.480
**SES**^e,i^											
	Low	24	(ref)				15		(ref)			
	Moderate-high	26	1.13	0.94	1.36	.187	13	0.88	0.63	1.23	.445
Friends who smoke		N/A^j^	1.23	1.13	1.34	<.001	N/A	1.04	0.93	1.16	.495
Household members who smoke		N/A	1.16	1.05	1.29	.005	N/A	1.22	1.00	1.50	.050
Internet, hours		N/A	1.05	1.01	1.09	.009	N/A	0.99	0.94	1.04	.697

^a^Percentages are weighted.

^b^Odds ratios are from weighted analyses.

^c^OR: odds ratio.

^d^Exposure=sometimes or often exposed.

^e^Based on postal code.

^f^ref: reference category.

^g^ads: advertisements.

^h^QLD: Queensland.

^i^SES: socioeconomic status.

^j^N/A: not applicable.

## Discussion

This study is the first to assess levels of exposure to online tobacco promotion in Australia, a notoriously “dark market.” The results suggest that not only is tobacco advertising and branding commonly encountered by young Australians, with almost a third of the youth surveyed in 2013 exposed, it is also increasing on social media, specifically Facebook.

Over the years of the study, exposure to online tobacco advertising and tobacco branding increased from 2010 to 2012. Concurrent changes to Australian tobacco advertising legislation included moving to retail tobacco displays bans and plain packaging of tobacco products. It has been noted that, as opportunities for tobacco promotion in one domain are restricted, the tobacco industry’s efforts in other domains increase [2]. In our study, the observed increases in exposure to online promotion were independent of increases in Internet use, or any changes in sample composition. These results may suggest that tobacco company efforts at attracting young Australians are being directed toward Internet-based advertising in the face of increasing restrictions on other forms of promotion, although this should be verified with monitoring of online advertising. Exposure to online advertising and branding appeared to plateau in 2013, concurrent with the national legislation banning Internet-based tobacco advertising originating from Australia. Nevertheless, and perhaps unsurprisingly given the borderless nature of the Internet, online tobacco promotions remained readily accessible to Australian youth.

This study extended previous research [19,29] by measuring exposure not only to tobacco advertisements, but also to tobacco company branding in general. While there was a degree of overlap between exposure to online tobacco advertising and tobacco branding, around 10% of youth reported being exposed to tobacco company branding in the absence of advertising. Of the participants who reported seeing tobacco branding online in 2013, around one-third reported seeing it in “traditional” Internet advertising formats such as pop-up advertisements, banner advertisements, sponsored search engine results, and website advertisements. This was a significant decrease from 2012, which might indicate a small effect of the national legislation introduced at the end of 2012.

Concurrent with the decrease in exposure to tobacco branding in traditional forms of online advertising, there were increases in exposure to branding on social media sites. Around a third of youth who saw online tobacco branding in 2013 reported seeing it on social media. Australians are prolific Facebook users, with 13.2 million users as of June 2014, making it one of the most popular websites in Australia [34]. It may be somewhat expected then that tobacco branding was most commonly reported as being seen on Facebook. Although Facebook prohibits advertisements that directly promote the sale of tobacco products, it does not prohibit advertisements that promote the use of tobacco products among like-minded individuals. Additionally, advertisements in this context are very narrowly defined, including only paid advertising that is purchased and prepared through the Facebook advertising portal. Any tobacco promotions appearing as unpaid content would be exempt from this policy. This presents a unique challenge for regulators, as unlike more traditional forms of Internet-based advertising, tobacco marketing on social media sites is less amenable to regulation and more difficult to directly attribute to tobacco companies [35]. Innovative approaches are likely to be required in order to determine the origins of this type of content, perhaps by engaging computer science and technology experts who can accurately navigate online networks.

There were also rapid increases in the proportion of youth seeing tobacco branding on YouTube. YouTube is also an exceptionally popular website, with an estimated 1 billion unique visitors from around the world every month [36]. Prosmoking imagery and tobacco promotions have been well documented on YouTube [14,37]. Again, like Facebook, YouTube does not allow tobacco products to be advertised on the site. The definition of advertising on YouTube is incredibly narrow, however, and only applies to *paid*forms of promotion on the site, such as advertisements embedded in popular videos or advertisements that appear for certain key word searches. British American Tobacco (BAT), for example, has its own YouTube channel, WelcomeToBAT, which includes videos outlining BAT’s public positions on harm reduction, illicit tobacco, marketing, and sustainable farming [38]. The broad definition of tobacco marketing outlined in Article 13 of the World Health Organization (WHO) Framework Convention on Tobacco Control (FCTC) could encompass this type of material because it has “the aim, effect or likely effect of promoting a tobacco product or tobacco use either directly or indirectly” [39].

In this study, participants most likely to recall seeing online tobacco advertising or branding were younger (12-15 years old) and/or female. Future research might explore potential reasons for this, including whether the tobacco industry is targeting younger people with media placement strategies, whether the advertising has been designed to appeal most to these demographics, or whether these groups are particularly sensitive to branding that speaks to evolving identities. Of note, nonsmoking youth were more likely to remember seeing tobacco advertising and branding than current smokers. Contrary to tobacco industry claims that any promotions are aimed at creating brand loyalty and switching among current smokers [40], online advertisements are reaching young people with no experience of smoking.

The fact that younger participants and nonsmokers were the most likely to report exposure to online tobacco advertising and branding is particularly concerning, as the younger never-smokers who remembered seeing tobacco advertising, promotions, or branding were more likely to be susceptible to smoking. This relationship was apparent even when controlling for smoking among family and friends. Building on the well-established link between exposure to tobacco company marketing and smoking susceptibility [2], this study is the first to establish a link between smoking susceptibility and exposure to online tobacco advertising as well as online tobacco branding of the type found on social media sites. We did not find an association between tobacco advertising or branding with smoking susceptibility for the older group of nonsmokers, indicating that other factors may be more important influencers of smoking susceptibility at that age.

### Strengths and Limitations

The strengths of this study include the collection of data over 4 years and in 2 states, resulting in a large and relatively representative sample. A wide range of covariates was used in all analyses, limiting the likelihood that observed changes in exposure over time were due to sample variations. Additionally, the inclusion of the mobile phone supplementary sample in 2013 allowed us to verify that the patterns of results we observed in the landline sample were primarily apparent in the dual-frame sample, reducing concerns about the use of sampling bias due to landline recruiting for 2010-2012. This study extends previous research on exposure of young people to online tobacco promotion by including exposure to tobacco branding as well as advertising and by identifying specific formats of branding encountered.

Limitations of the study include relying on self-reported exposure to online material—it can be difficult to remember where precisely something was seen online, as evidenced by the high number of participants stating that they did not know where they encountered tobacco branding. Given that much of what we see online is unlikely to be recalled, we are potentially underestimating rates of actual exposure. The findings of this study should therefore be interpreted alongside existing investigations about the number and nature of advertisements and promotions, particularly on social media sites [14]. Additionally, young people have been known to underreport smoking-related behaviors over the phone compared with when they self-complete a survey [41]—this might have slightly diminished estimated rates of smoking and smoking susceptibility, particularly in the younger age group, but this effect would have been consistent across years. Finally, the observed association between exposure to online tobacco promotion and smoking susceptibility is cross-sectional in nature, and longitudinal data would be needed to investigate the order of effects. However, the inclusion of a large number of appropriate covariates demonstrates that this association exists independently of the influence of the exposure of young people to peer and family smoking.

### Conclusions

The relatively common experience of exposure to online tobacco advertising, promotion, and branding among Australian youth reinforces the importance of comprehensive restrictions on Internet tobacco promotion, as well as strong counter-advertising initiatives. The WHO FCTC recognizes that cross-border promotions are a threat to domestic laws that ban tobacco advertising [42]. This is particularly true for online promotions where the borderless nature of the Internet will require cooperation among parties to the WHO FCTC in order for tobacco advertising bans to be effective. Establishing mechanisms where WHO FCTC parties can monitor, report, and act on promotions that leak across borders is paramount. Even in such a climate of cooperation, it is likely that the greatest challenge in monitoring and enforcing restrictions on Internet tobacco promotions will be to linking such promotions to the tobacco industry, especially on social media. Accordingly, an expert advisory group has been proposed to keep WHO FCTC parties up to date on relevant developments in technology in cross-border tobacco advertising, promotion, and sponsorship, and in best practices for responding to these forms of promotion [43]. There is also a need to continually assess the media strategies used in counter-advertising so that antitobacco messages are reaching young people in the digital spaces where they are likely to be encountering tobacco promotion.

Our results stress the need for continued research and surveillance of tobacco marketing that is penetrating new and underregulated digital media. In order to continue downward trends in smoking and the denormalization of smoking among youth, online advertising and promotions need to be subject to more comprehensive restrictions. There is a misconception that online advertising is a weaker or less penetrative form of marketing [44] and is simply used to augment more traditional offline media promotions. Evidence from the alcohol control field demonstrates that online advertising reduces the effectiveness of regulations banning offline advertising because online advertising replaces, rather than simply complements, offline advertising [45]. Given the demonstrated effect of tobacco promotion on tobacco uptake by young people [24], increased efforts to restrict youth exposure to tobacco promotion through these new media outlets are critical.

## Abbreviations

BAT: British American Tobacco
FCTC: Framework Convention on Tobacco Control
NSW: New South Wales
OR: odds ratio
SES: socioeconomic status
TPIS: Tobacco Promotion Impact Study
WHO: World Health Organization
